# Loss of DNA methylation is not associated with venetoclax–hypomethylating agents synergy in acute myeloid leukaemia cells

**DOI:** 10.1111/bjh.70729

**Published:** 2026-07-27

**Authors:** Aida Selimović‐Pašić, Lisa Haglund, Maike Bensberg, Júlia Goldmann, Padraig D'arcy, Sandra Hellberg, Colm E. Nestor

**Affiliations:** ^1^ Division of Children's and Women's Health, Department of Biomedical and Clinical Sciences, Faculty of Medicine and Health Sciences Linköping University Linköping Sweden; ^2^ Division of Clinical Chemistry and Pharmacology, Faculty of Medicine and Health Sciences Linköping University Linköping Sweden; ^3^ Science for Life Laboratory, Linköping University Linköping Sweden

**Keywords:** acute myeloid leukaemia, DNA damage, DNA methylation, drug synergy, hypomethylating agents, venetoclax

To the Editor,

Haematological malignancies are characterized by abnormal cell survival driven by overexpression of anti‐apoptotic proteins like BCL‐2, promoting disease progression and therapeutic resistance.^1^ In acute myeloid leukaemia (AML), the BCL‐2 inhibitor venetoclax (VEN) has shown limited clinical efficacy as monotherapy.^2^ However, its combination with hypomethylating agents (HMAs) such as azacitidine (AZA) or decitabine (DAC) significantly improves responses and survival in elderly AML patients who are ineligible for intensive chemotherapy.^3^, ^4^, ^5^ Nonetheless, toxicities and frequent relapses remain major challenges,^6^, ^7^ underscoring the need for a deeper understanding of the cellular and molecular dynamics of these combination therapies to optimize treatment strategies.

Preclinical studies suggest VEN and HMAs act synergistically, potentially mediated by both epigenetic mechanisms, such as DNA demethylation that activates cell death pathways and upregulates pro‐apoptotic genes,^8^, ^9^ and non‐epigenetic effects such as DNA damage.^10^ However, the combined DNA demethylating effects and DNA damage response of conventional HMAs have made it challenging to determine which mechanism contributes to this synergy.^11^ We set out to clarify the relative contributions of DNA demethylation and DNA damage‐related cytotoxicity to the observed synergy between VEN and HMAs, using the novel DNA methyltransferase 1 (DNMT1) selective inhibitor GSK‐3685032 (GSK5032) (Figure 1A).^12^ With potent DNA demethylating activity and limited cytotoxicity through DNA damage,^13^ GSK5032 serves as a useful tool to investigate the contribution of these effects to antitumor efficacy. Here, we used human AML cell lines as a controlled, reproducible model to examine the relative roles of DNA demethylation and DNA damage–associated effects in venetoclax‐HMA responses.

**FIGURE 1:**
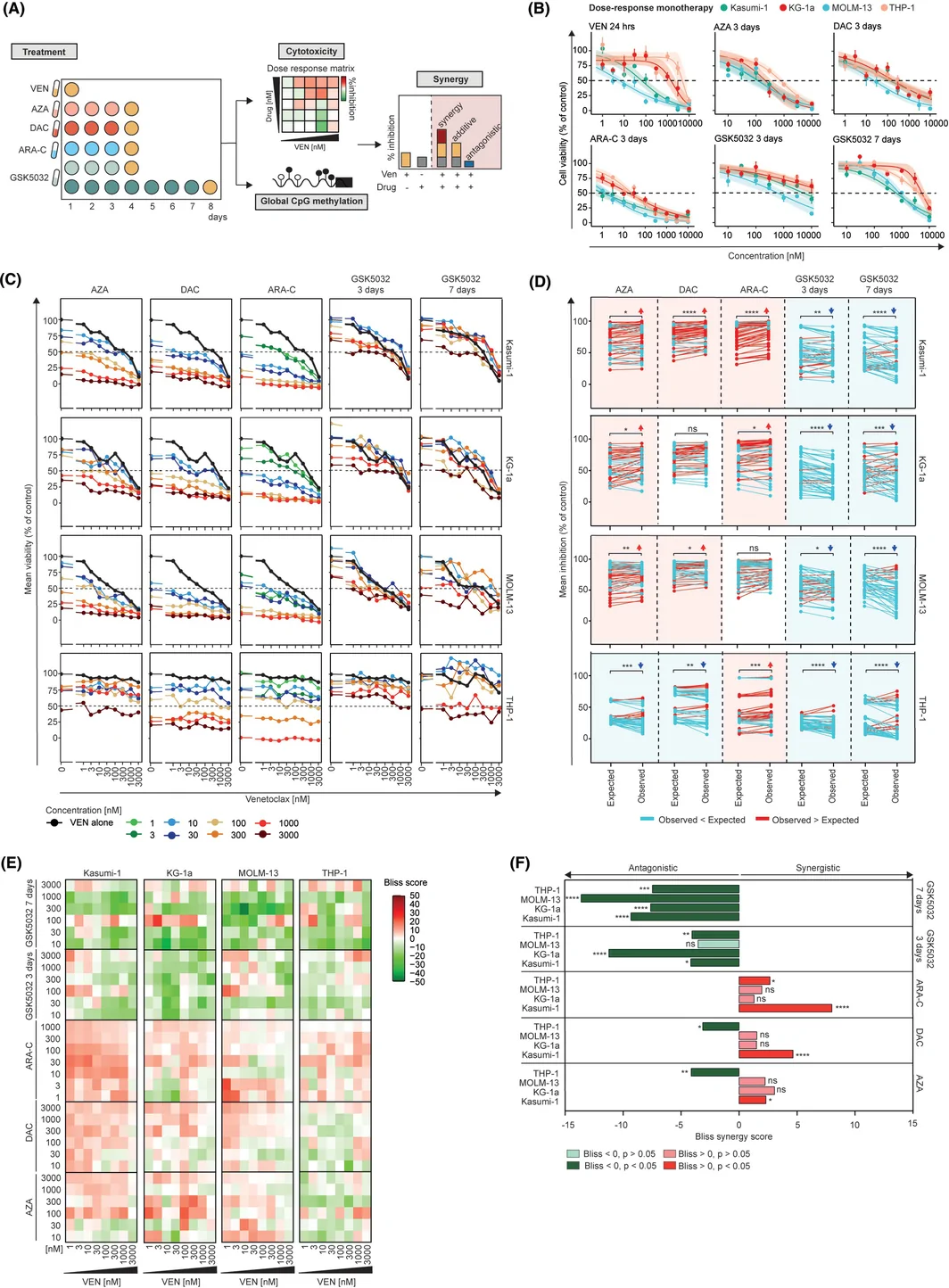
Lack of synergistic effect between a non‐DNA‐damaging DNA demethylating agent and venetoclax in AML cell lines. (A) Overview of the experimental design: Four AML cell lines were treated with increasing concentrations of azacitidine (AZA), decitabine (DAC), GSK‐3685032 (GSK5032) or cytarabine (ARA‐C) for 3 days (or 7 days for GSK5032), followed by 24‐h venetoclax (VEN) treatment. Treatment responses were assessed by Alamar Blue assay and whole‐genome DNA methylation sequencing. Drug–VEN synergy was evaluated using Bliss and ZIP synergy models. (B) Monotherapy dose–response curves for all four AML cell lines following treatment with varying concentrations of AZA, DAC, ARA‐C, GSK5032 for 3 days (and after 7 days for GSK5032) or VEN for 24 h. Cell viability was assessed by Alamar Blue assay. Mean ± SEM of triplicates are shown. (C) Mean viability (% of control) following combination therapy with VEN and AZA, DAC, ARA‐C or GSK5032 after 3 days (and 7 days for GSK5032). Black lines indicate VEN monotherapy. (D) Comparison of observed combination effects to the expected effects calculated using the Bliss model. Lines connect the expected and observed values for each dose combination; red indicates potential synergy and blue antagonism. Statistical differences between the expected and observed effects were assessed using the Wilcoxon rank sum test. (E) Bliss synergy scores for VEN combinations with AZA, DAC, ARA‐C or GSK5032, show as heatmaps (three replicates per condition). (F) Mean Bliss synergy score and significance for each VEN combination (AZA, DAC, ARA‐C, GSK5032) divided per cell line. *p < 0.05, **p < 0.01, ***p < 0.001 and ****p < 0.0001.

Initial drug‐sensitivity assays were performed to evaluate the effects of AZA, DAC, GSK5032 and VEN as single agents in four AML cell lines. To separate DNA damage from demethylation effects, we also included cytarabine (ARA‐C), a nucleoside analogue that induces DNA damage without demethylating activity. MOLM‐13 and Kasumi‐1 were more sensitive to VEN, while KG1‐a and THP‐1 appeared more resistant in line with previous studies (Figure 1B, Figure S1A–C).^14^, ^15^ GSK5032 displayed lower toxicity at both three and 7 days as compared to AZA and DAC, whereas ARA‐C treatment induced the highest cytotoxicity in all cell lines (Figure 1B, Figure S1C).

Since VEN has previously been shown to have synergistic effects with HMAs,^8^, ^9^, ^10^ we proceeded to determine whether similar synergistic effects could be observed using the DNA demethylating drug GSK5032. The treatment schedule was adapted for cell line experiments to allow for sufficient DNA demethylation prior to VEN exposure, thereby enabling the evaluation of potential synergistic effects between DNA demethylation and BCL‐2 inhibition. All AML cell lines were treated with varying concentrations of AZA, DAC, GSK5032 or ARA‐C for 3 or 7 days, followed by a 24‐h treatment with increasing concentrations of VEN, generating a comprehensive dose–response matrix of at least 63 different treatment combinations for synergy analysis. VEN combined with AZA, DAC or ARA‐C reduced cell viability compared to VEN alone across nearly all tested concentrations (Figure 1C, Figures S2–S5). Interestingly, while several dose combinations of VEN‐GSK5032, particularly those containing higher concentrations of GSK5032, reduced cell viability compared to VEN monotherapy, lower concentrations of GSK5032 (10–100 nM) did not lead to higher cytotoxicity. In contrast, AZA, DAC and ARA‐C reduced cell viability at even lower concentrations (Figure 1C). Notably, the observed inhibition of GSK5032‐VEN was significantly lower than the expected effect, calculated using Bliss Independence model, which assumes the two drugs act independently based on their monotherapy responses (Figure 1D). In comparison, combinations with AZA, DAC and ARA‐C frequently resulted in significantly greater inhibition than expected for purely independent drug effects, suggesting potential synergy (Figure 1D). Corresponding synergy scores confirmed that VEN‐AZA and VEN‐DAC combinations were more synergistic compared to VEN‐GSK5032 (Figure 1E, Figure S6A–D, Table S1). To assess the robustness of the synergy across different reference models, we also evaluated synergy using the zero interaction potency (ZIP) synergy model, which showed broadly consistent results with Bliss. Both models showed that VEN‐AZA and VEN‐DAC combinations are more synergistic than VEN‐GSK5032 (Figures S7 and S8). Interestingly, VEN and GSK5032 more often exhibited antagonistic responses across all tested combinations (Bliss score <0; Figure 1E, Table S1) and across all cell lines, particularly after 7 days of treatment with GSK5032 (Figure 1F, Figure S6A–D, Table S2). Since we observed no synergistic effect for GSK5032 but high synergy for ARA‐C combined with VEN (Figure 1F), it could be hypothesized that the observed effects seen for AZA and DAC are not solely attributable to DNA demethylation and may involve additional mechanisms beyond DNMT1 inhibition.

Given the notably weaker or non‐synergistic effects observed with GSK5032 compared to all other treatments, we proceeded to validate the DNA demethylating activity of all four drugs using our low‐coverage whole methylome sequencing approach^13^ to assess global methylation levels after treatment (Figure 2A, Tables S3 and S4). Global DNA methylation levels obtained with this approach reflected methylation at long interspersed nucleotide elements (LINEs), which have long been used as a proxy for global methylation levels (Figure 2B, Table S5), validating the reliability of our approach. As expected, and in line with its mode of action, ARA‐C did not affect DNA methylation levels (Figure 2C), suggesting that the synergistic effects observed with VEN are due to the DNA‐damaging properties of AZA, DAC and ARA‐C. The greatest loss of DNA methylation was achieved with GSK5032, with as little as 15%–26% of methylation remaining in Kasumi‐1 and MOLM‐13 after 7 days treatment (Figure 2C). This loss of DNA methylation corresponded with increased expression of the DNA methylation‐sensitive genes, Deleted in Azoospermia‐Like (*DAZL*) and G antigen 12G (*GAGE*), as assessed by qPCR (Figure S9). Importantly, these were also the cell lines that had the lowest Bliss synergy scores at 7 days (Kasumi‐1: mean Bliss score −9.32, *p* = 3.3e‐07; MOLM‐13: −13.62, *p* = 2.3e‐11; Figure S6A,C), suggesting that loss of DNA methylation alone does not correlate with increased venetoclax synergy.

**FIGURE 2:**
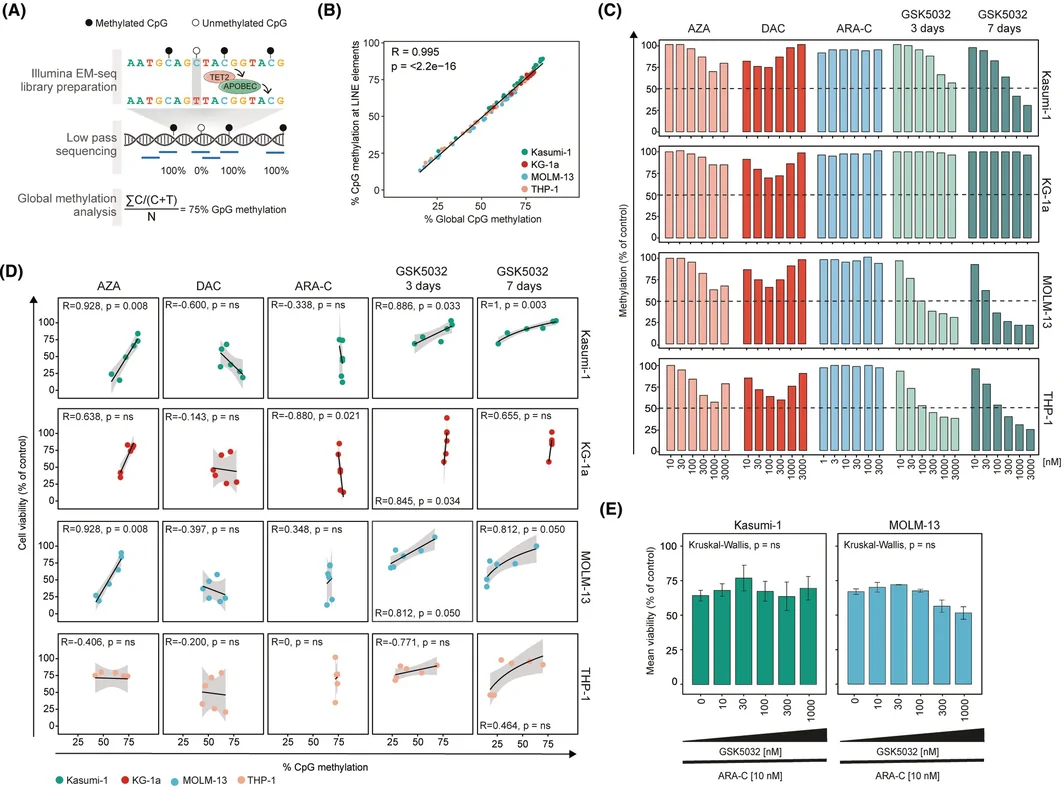
DNA demethylation does not enhance cytotoxicity. (A) Schematic overview of the enzymatic methyl‐sequencing (EM‐seq) workflow. EM‐seq library preparation involves the oxidation of methylated and hydroxymethylated cytosines by tet methylcytosine dioxygenase 2 (TET2), followed by the deamination of unmethylated cytosines to uracil by APOBEC, which is subsequently read as thymine during sequencing. Libraries are sequenced at low coverage using paired‐end sequencing. Global CpG methylation levels were calculated as ∑*C*/(*C* + *T*)/*N*, where *C* represents the number of methylated CpG sites, *T* the number of unmethylated CpG sites, and N the total number of CpG sites analysed. (B) Correlation between average global CpG methylation assessed by low‐coverage whole methylome sequencing and DNA methylation at long interspersed nucleotide elements (LINEs) across all cell lines. Correlation was done with Spearman's rank correlation. (C) Global CpG methylation relative to control in Kasumi‐1, KG‐1a, MOLM‐13 and THP‐1 cells after treatment with increasing concentrations of azacitidine (AZA), decitabine (DAC), cytarabine (ARA‐C) or GSK‐3685032 (GSK5032) for 3 days (and 7 days for GSK5032). (D) Relationship between global CpG methylation and cell viability for each treatment and cell line. Generalized linear regression models with 95% confidence intervals are shown. Correlations were assessed using Spearman's rank correlation; coefficients and *p*‐values are indicated (ns denoted *p* > 0.05). (E) Mean viability after treatment with increasing concentrations of GSK5032 for 3 days and fixed concentration of ARA‐C in Kasumi‐1 and MOLM‐13. Kruskal–Wallis was used to test for statistical differences.

Global methylation levels in Kasumi‐1 and MOLM‐13 cells significantly correlated with cell viability following treatment with AZA and GSK5032 (Figure 2D). After 7 days of GSK5032 treatment in Kasumi‐1 cells, global methylation decreased to 26%, but cell viability remained high at 69%. In contrast, treatment with AZA for 3 days resulted in 66% global methylation, with only 15% cell viability (Figure 2D, Table S6). Additionally, the two drugs showed markedly different synergistic effects in combination with VEN (Figure 1F). GSK5032, which induced the strongest demethylation but had relatively limited cytotoxicity, showed minimal synergy with VEN. In contrast, AZA, which had more modest demethylating effects but greater cytotoxicity, was strongly synergistic with VEN. This suggests that DNA damage‐related cytotoxic potency, rather than the extent of DNA demethylation, could contribute to the differential synergistic responses observed with VEN.

To explore whether the DNA‐demethylating and DNA damage‐inducing properties of HMAs act together to induce toxicity, we combined GSK5032 (10–1000 nM) with a fixed concentration of ARA‐C (10 nM). Because the GSK5032 concentrations used induce robust DNA demethylation with relatively limited effect on cell viability, this experiment allowed us to assess the combined effects of DNA demethylation and DNA damage on cytotoxic responses. Cell viability was not significantly altered by the addition of increasing concentrations of GSK5032 compared to ARA‐C alone (Figure 2E). A slight trend towards decreased viability was observed in the MOLM‐13 cell line, consistent with the modest cytotoxicity of GSK5032 monotherapy in these cells (Figure 1B). Overall, these findings show that GSK5032 does not enhance ARA‐C‐mediated cytotoxicity, implying that DNMT1 inhibition alone is not sufficient to increase cytotoxic responses under these conditions. Lastly, to assess whether potential long‐term demethylation‐dependent effects could influence VEN sensitivity, VEN‐resistant THP‐1 cells were pre‐treated with a demethylating concentration of GSK5032 for 72 h and subsequently cultured for 14 days before exposure to VEN. This experimental design was used to allow GSK5032‐induced DNA demethylation to persist over time and potentially sensitize cells to subsequent VEN treatment. Although lower cell densities were more sensitive to both GSK5032 and VEN, pre‐treatment with GSK5032 did not enhance response to VEN (Figure S10).

Taken together, the observed synergistic effects are consistent with the known mechanisms of venetoclax and nucleoside analogues, which target apoptotic regulation and induce DNA‐damage activation response pathways respectively. A trend towards antagonism between VEN and conditions associated with more pronounced loss of DNA methylation was observed in several cell lines, suggesting that DNA methylation changes alone do not determine venetoclax combination sensitivity. These findings underscore the importance of understanding drug interactions to better optimize and refine venetoclax‐based combination therapies. Our study focuses on the short‐term cytotoxic responses and provides a systematic comparison of venetoclax combination across epigenetic and DNA‐damaging treatments. While this experimental design captures acute pharmacological interactions, the current setup does not fully resolve potential longer term effects associated with epigenetic reprogramming. Further studies using primary cells and in vivo models will be important to assess the relevance of these findings in more clinically relevant settings.

## AUTHOR CONTRIBUTIONS

**Padraig D'arcy:** Methodology; conceptualization; writing – review and editing. **Aida Selimović‐Pašić:** Methodology; formal analysis; writing – review and editing; writing – original draft; investigation; conceptualization; visualization; validation. **Júlia Goldmann:** Investigation; writing – review and editing. **Maike Bensberg:** Writing – review and editing; methodology; investigation. **Sandra Hellberg:** Writing – original draft; writing – review and editing; visualization; formal analysis; data curation; project administration; supervision; investigation; software. **Colm E. Nestor:** Conceptualization; funding acquisition; writing – original draft; writing – review and editing; project administration; supervision; resources. **Lisa Haglund:** Investigation; writing – original draft; writing – review and editing; visualization; methodology; formal analysis; data curation; validation; software.

## FUNDING INFORMATION

Ths work was funded by the Joanna Cocozza Foundation, the Linköping University Cancer Network, the Swedish Cancer Society (project 201231 PJF) and the Swedish Childhood Cancer Fund (PR2022‐0121) to C.E.N.

## CONFLICT OF INTEREST STATEMENT

The authors declare no conflict of interest.

## ETHICS STATEMENT

This work included commercially available cell lines that do not require ethical approval from the Ethical Review Board.

## Supporting information


**Data S1.** Supporting information S1.

**Figure S1.** (A) Monotherapy dose–response curves for all four AML cell lines following treatment with increasing concentrations of venetoclax (VEN) for 48 h or azacitidine (AZA) and decitabine (DAC) for 7 days. Cell viability was assessed by Alamar Blue assay. Mean and standard error of the mean of triplicates for each cell line are shown. (B) Overview of statistical difference between cell lines for the different treatment. *p*‐values were computed using Wilcoxon rank sum tests and adjusted using Benjamini–Hochberg. Significantly adjusted *p*‐values are highlighted in red. (C) Monotherapy dose–response curves for treatment with increasing concentrations of VEN for 24 h, AZA, DAC, cytarabine (ARA‐C) and GSK‐3685032 (GSK5032) for 3 days (and 7 days for GSK5032) in all four AML cell lines. Cell viability was determined using Alamar Blue assay. Mean and standard error of the mean from triplicate measurements are shown for each treatment and concentration.
**Figure S2.** (A–E) Dose–response matrices for VEN‐AZA, VEN‐DAC, VEN‐ARA‐C and VEN‐GSK5032 in Kasumi‐1 cells. AZA, azacitidine; ARA‐C, cytarabine; DAC, decitabine; GSK5032, GSK‐3685032; VEN, venetoclax.
**Figure S3.** (A–E) Dose–response matrices for VEN‐AZA, VEN‐DAC, VEN‐ARA‐C and VEN‐GSK5032 in KG‐1a cells. AZA, azacitidine; ARA‐C, cytarabine; DAC, decitabine; GSK5032, GSK‐3685032; VEN, venetoclax.
**Figure S4.** (A–E) Dose–response matrices for VEN‐AZA, VEN‐DAC, VEN‐ARA‐C and VEN‐GSK5032 in MOLM‐13 cells. AZA, azacitidine; ARA‐C, cytarabine; DAC, decitabine; GSK5032, GSK‐3685032; VEN, venetoclax.
**Figure S5.** (A–E) Dose–response matrices for VEN‐AZA, VEN‐DAC, VEN‐ARA‐C and VEN‐GSK5032 in THP‐1 cells. AZA, azacitidine; ARA‐C, cytarabine; DAC, decitabine; GSK5032, GSK‐3685032; VEN, venetoclax.
**Figure S6.** (A–D) 3D surface plots depicting synergy scores for the combination effects of VEN‐AZA, VEN‐DAC, VEN‐ARA‐C and VEN‐GSK5032 across all four AML cell lines. Synergy scores were calculated using the Bliss Independence model. Mean Bliss score and the corresponding p‐values are shown above each plot. AZA, azacitidine; ARA‐C, cytarabine; DAC, decitabine; GSK5032, GSK‐3685032; VEN, venetoclax.
**Figure S7.** (A) Comparison of observed combination effects to the expected effects calculated using the ZIP synergy model. Lines connect the expected and observed values for each dose combination; red indicates potential synergy and blue potential antagonism. Statistical differences between the expected and observed effects were assessed using the Wilcoxon rank sum test. (B) ZIP synergy scores for VEN combinations with AZA, DAC, ARA‐C or GSK5032, show as heatmaps (three replicates per condition). (C) Mean ZIP synergy score and significance for each VEN combination (AZA, DAC, ARA‐C, GSK5032) divided per cell line. *p < 0.05, **p < 0.01, ***p < 0.001 and ****p < 0.0001. AZA, azacitidine; ARA‐C, cytarabine; DAC, decitabine; GSK5032, GSK‐3685032; VEN, venetoclax.
**Figure S8.** (A–D) 3D surface plots depicting synergy scores for the combination effects of VEN‐AZA, VEN‐DAC, VEN‐ARA‐C and VEN‐GSK5032 across all four AML cell lines. Synergy scores were calculated using the ZIP synergy model. Mean ZIP score and the corresponding *p*‐values are shown above each plot. AZA, azacitidine; ARA‐C, cytarabine; DAC, decitabine; GSK5032, GSK‐3685032; VEN, venetoclax.
**Figure S9.** Expression of the methylation‐sensitive genes *DAZL* and *GAGE12* was measured by qPCR in KG‐1a and MOLM‐13 cells after treatment with increasing concentrations of azacitidine (AZA), decitabine (DAC) and GSK‐3685032 (GSK5032) for 3 days. Relative differences in expression compared to the housekeeping gene *GAPDH*.
**Figure S10.** Viability of THP‐1 cells following GSK‐3685032 (GSK5032) pre‐treatment and venetoclax (VEN) exposure. THP‐1 cells were pretreated with 100 nM GSK5032 or DMSO for 72 h, then seeded at 10, 100 or 1000 cells per well and cultured for 14 days. Cells were subsequently treated with 10 nM venetoclax (VEN) or DMSO for 24 h. Viability was assessed using the Alamar Blue assay. The bars represent mean of six replicate wells per condition.

**Table S1.** Synergy scores for the combination treatments with venetoclax (VEN) and azacitidine (AZA), decitabine (DAC), cytarabine (ARA‐C) for 3 days or GSK‐3685032 (GSK5032) for three and 7 days in Kasumi‐1, KG‐1a, MOLM‐13 and THP‐1. Synergy scores were calculated using the Bliss Independence model.

**Table S2.** Summary statistics of the synergy scores in Kasumi‐1, KG‐1a, MOLM‐13 and THP‐1.

**Table S3.** Sequencing metrics for low pass whole methylome sequencing of Kasumi‐1, KG‐1a, MOLM‐13 and THP‐1 after treatment with increasing concentrations of azacitidine (AZA), decitabine (DAC), cytarabine (ARA‐C) for 3 days or GSK‐3685032 (GSK5032) for 3 and 7 days.

**Table S4.** Average CpG methylation as percentage, globally and at LINE elements, assessed by low pass whole methylome sequencing of Kasumi‐1, KG‐1a, MOLM‐13 and THP‐1 after treatment with increasing concentrations of azacitidine (AZA), decitabine (DAC), cytarabine (ARA‐C) for 3 days or GSK‐3685032 (GSK5032) for 3 and 7 days.

**Table S5.** Average coverage per CpG, assessed by low pass whole methylome sequencing of Kasumi‐1, KG‐1a, MOLM‐13 and THP‐1 after treatment with increasing concentrations of azacitidine (AZA), decitabine (DAC), cytarabine (ARA‐C) for 3 days or GSK‐3685032 (GSK5032) for 3 and 7 days.

**Table S6.** Average mean viability and global CpG methylation for Kasumi‐1, KG‐1a, MOLM‐13 and THP‐1 after treatment with increasing concentrations of azacitidine (AZA), decitabine (DAC), cytarabine (ARA‐C) for 3 days or GSK‐3685032 (GSK5032) for 3 and 7 days.

## Data Availability

The low‐coverage genome‐wide methylation sequencing data is available at ArrayExpress under accession E‐MTAB‐14969.
