# Supplementary material for: Loss of DNA methylation is not associated with venetoclax–hypomethylating agents synergy in acute myeloid leukaemia cells
Source: Br J Haematol. 2026 Jul 27;209(4):1907–12. doi: 10.1111/bjh.70729 (PMC13674118; doi:10.1111/bjh.70729)
Supplement: Supplementary file 1 [file BJH-209-1907-s001.docx]

**Methods**

*Cell lines*

AML cell lines MOLM-13, Kasumi-1, KG-1a and THP-1 were ordered from German Collection of Microorganisms and Cell Cultures (DSMZ) and kept in humidified incubator at 37°C with 5% CO_2_. Cells were cultured in American Type Culture Collection (ATCC)-modified RPMI 1640 medium (Thermo Fisher Scientific, A10491), supplemented with 10% heat-inactivated fetal bovine serum (FBS) (Thermo Fisher Scientific, 11550356, 17593595) and 1% penicillin-streptomycin (100 U/mL penicillin, 100 μg/mL streptomycin) (Thermo Fisher Scientific, 15140). Cells were passaged every two or three days and routinely checked for mycoplasma contamination using the MycoAlert Mycoplasma Detection Kit (Lonza, LT07-318).

*Treatment with drugs*

5-azacytidine (AZA; Sigma-Aldrich, A1287), decitabine (DAC; Sigma-Aldrich, A3656), GSK-3685032 (GSK5032; MedChemExpress, HY-139664) and Venetoclax (VEN; MedChemExpress, HY-15531) stock solutions were dissolved in Dimethyl Sulfoxide (DMSO) (Sigma-Aldrich, D8418), while Cytarabine (ARA-C; Sigma-Aldrich, C1768) was dissolved in Ultrapure H_2_O (Millipore, Milli-Q) to a final concentration of 10 mM. Stocks were kept at -20°C for up to one month. 1.5 × 10⁵ cells in 500 µl RPMI medium were treated under three experimental conditions. For monotherapy, cells received VEN for 24 h and 48 h; AZA, DAC, GSK5032, or ARA-C for 3 days; or GSK5032 for 7 days. For combination treatment, cells were treated with AZA, DAC, GSK5032, or ARA-C for 3 days, followed by VEN for 24 h (**Figure 1A**). For the ARA-C/GSK5032 co-treatment, Kasumi-1 and MOLM-13 cells were exposed to a fixed concentration of ARA-C (10 nM) together with increasing concentrations of GSK5032 for 3 days.

To evaluate whether GSK5032 pretreatment sensitized cells to VEN in the long term, THP-1 cells were treated with 100 nM GSK5032 for 72 h. Following treatment, cells were harvested and seeded at densities of 10, 100, or 1000 cells per well in 96-well plates. Cells were cultured for 14 days and subsequently exposed to 10 nM VEN for 24 h. Relative cell numbers were then assessed using the Alamar Blue assay as described below. In all treatment settings, drugs were refreshed every 24 h. Untreated controls were exposed to equivalent concentrations of DMSO or ultrapure H₂O.

*Alamar Blue cell viability assay*

Following treatment, 10 µl of Alamar Blue cell viability reagent (Thermo Fisher Scientific, DAL1100) was mixed with 90 µl of cell suspension in 96-well plate. Absorbance was measured on a plate reader (Tecan, Spark10M) at 570 nm and 600 nm in three or six technical replicate wells per condition. Cell viability was determined by measuring reduced Alamar blue, which reflects cellular metabolic activity, and was normalized to untreated controls according to the manufacturer’s instructions. Dose response curves were generated in R (version 4.4.2) with the *drc* package version 3.0-1 fitting a three-parameter log-logistic function the *fct = LL.3()*.

*Synergism analysis*

The expected combination effect of VEN with AZA, DAC, ARA-C or GSK5032 was calculated using the Bliss Independence model: $y_{Bliss}=y_{1}+y_{2}- {(y}_{1}{* y}_{2})$ , where $y_{1}$ and $y_{2}$represent the fractional inhibition effects of the single-agent treatments (VEN and the respective partner drug). Both the expected combination effects and synergy scores were calculated using the Bliss Independence model as implemented in SynergyFinder Plus R package (version 3.14.0).^1^ Due to the nature of AlamarBlue analysis, where values from untreated control wells is subtracted from treated samples, calculated viability values can fall below 0% or exceed 100%, depending on the treatment effect. All values were therefore adjusted prior to analysis to constrain all values within the biologically relevant range of 0-100%; values below 0% were set to 0%, and those above 100% were set to 100%. Data was formatted using *ReshapeData()* including *noise = FALSE* and *data_type = “viability”* and *CalculateSynergy()* was used to compute synergy using *method = “Bliss”* with default parameters. Synergy scores of >0 were regarded as synergistic.

*DNA and RNA isolation*

DNA and RNA were extracted using the Quick-DNA/RNA Kits (Zymo Research, D7001 and D7003) according to the manufacturer's instructions, with additional in-column RNase/DNase treatments. DNA samples were treated with 4 U RNase A/T1 (Thermo Fisher Scientific, EN0551), and RNA samples with 5 U DNase I (Zymo Research, E1010), each for 15 minutes at room temperature. DNA and RNA concentrations were measured using a NanoDrop 2000 spectrophotometer (Thermo Fisher Scientific).

*Quantitative PCR*

200 ng RNA was reversed transcribed into cDNA using the High-Capacity cDNA Reverse Transcription Kit (Applied Biosystems, 4368814), according to the manufacturer’s instructions. The reverse transcription reaction was carried out in a SimpliAmp™ Thermal Cycler (Thermo Fisher Scientific) with an initial incubation at 37°C for 2 hours, followed by a 5-minute inactivation step at 85°C. cDNA was diluted 1:10 to a total volume of 200 μl. For each PCR reaction, 4.5 μl of diluted cDNA was combined with 5 μl of TaqMan™ Fast Universal PCR Master Mix (Thermo Fisher Scientific, 4352042) and 0.5 μl of TaqMan probes (Thermo Fisher Scientific) for the following genes: *DAZL* (Hs00154706_m1), *GAGE12* (Hs04190947_gH), and *GAPDH* (Hs02758991_g1). qPCR was performed in technical duplicates on a QuantStudio™ 7 Flex Real-Time PCR System (Thermo Fisher Scientific) using the following thermal cycling conditions: 95°C for 20 seconds and 40 cycles of 95°C for 1 second and 60°C for 20 seconds. Expression of the known DNA methylation-sensitive genes *DAZL* and *GAGE12* quantified using the ΔCt method and normalized to *GAPDH* expression. HMAs induced expression of both *DAZL* and *GAGE12* indicating local transcriptional changes to loss of DNA methylation (**Supplemental Figure 9**).

*Library preparation for enzymatic methyl-sequencing*

200 ng genomic DNA, 0.001 ng pUC19 (methylated), and 0.02 ng lambda (unmethylated) DNA was diluted in 0.1X TE buffer, to a final volume of 50 µl. DNA was sheared using the Covaris S220 ultrasonicator, yielding an average fragment size of 302 base pairs. Library preparation was carried out using the NEBNext Enzymatic Methyl-seq Kit (New England Biolabs, E7120S/L) following the manufacturer's standard library protocol. Briefly, following DNA end repair and adaptor ligation, methylated and hydroxymethylated cytosines were oxidized using TET2. The DNA was then denatured by heating in formamide at 85 °C for 10 minutes, followed by the deamination of unmethylated cytosines to uracil by APOBEC, which is read as thymine during sequencing. Libraries were PCR-amplified for four cycles. Final library concentration was determined by the Qubit 1X dsDNA HS assay (Thermo Fisher Scientific, Q33231), and library size was evaluated on a Fragment Analyzer or 4150 TapeStation with the DNF-474 HS NGS Fragment Kit or D1000 kit (Agilent), respectively. The libraries were sequenced using low-coverage whole methylome sequencing with 150 bp paired-end reads, generating an average of 232,533 read pairs per sample (min 118,415 reads, max 453,764 reads). The sequencing was performed on the MiSeq machine (Illumina) using the MiSeq Reagent Kit v2 (300 cycles) (Illumina). Only samples with CHG and CHH methylation levels < 1.5 % were included in downstream analysis to confirm successful library conversion.

*Global methylation analysis from low-coverage whole-genome enzymatic methyl-sequencing sequencing*

Reads were trimmed with Trim Galore version 0.6.10 (<https://github.com/FelixKrueger/TrimGalore>) using default parameters including *--paired* and *--fastqc*. Sequences for pUC19 (M77789.2) and lambda (J02459.1) were added to the human hg38 reference genome (GCA_000001405.15_GRCh38_no_alt_analysis_set). Trimmed reads were aligned (bwameth.py) to the indexed genome (*bwameth.py index*) with bwa-meth version 0.2.7 (<https://github.com/brentp/bwa-meth> arXiv:1401.1129v2) with default parameters. Aligned reads were converted to bam format (*samtools view*) and sorted (*samtools sort*) using samtools version 1.13.^2^ Duplicate reads were marked with Picard *MarkDuplicates* [(http://broadinstitute.github.io/picard](http://broadinstitute.github.io/picard)) with default parameters and files were indexed with *samtools index*. DNA methylation was called with *MethylDackel extract* (version 0.6.1[, https://github.com/dpryan79/MethylDackel](https://github.com/dpryan79/MethylDackel)) with trimmed reads by 5 bases from each end (*--nOT 6,5,6,5* and *--nOB 6,5,6,5*). Non-CpG methylation was extracted (*--CHG* and *--CHH*) to evaluate conversion. For CpG methylation calls, strand-specific information was merged (*MethylDackel extract* *–mergeContext --nOT 6,5,6,5 --nOB 6,5,6,5*). Additionally, the number of reads covering each CpG was extracted with *MethylDackel extract* *(--nOT 6,5,6,5; --nOB 6,5,6,5; --mergeContext, --counts*). From the strand-merged CpG methylation calls, global average CpG methylation levels were calculated after excluding both control DNAs (pUC19: M77789.2; lambda: J02459.1) and mitochondrial DNA (chrM) (**Supplementary Table 3**).

*Data visualization*

Data was visualized using ggplot2 (for line graphs, heatmaps, and bar graphs; version 3.5.1). Statistical analysis was conducted in R (version 4.4.2) as described if not stated otherwise.

**Code availability**

All code used for data analysis is available at <https://github.com/ColmNestorLab/Synergy>.

**References**

**1**. Zheng S, Wang W, Aldahdooh J, et al. SynergyFinder Plus: Toward Better Interpretation and Annotation of Drug Combination Screening Datasets. *Genomics Proteomics Bioinformatics*. 2022;20(3):587-596.

**2**. Li H, Handsaker B, Wysoker A, et al. The Sequence Alignment/Map format and SAMtools. *Bioinformatics*. 2009;25(16):2078-2079.
