# Supplementary material for: Loss of DNA methylation is not associated with venetoclax–hypomethylating agents synergy in acute myeloid leukaemia cells
Source: Br J Haematol. 2026 Jul 27;209(4):1907–12. doi: 10.1111/bjh.70729 (PMC13674118; doi:10.1111/bjh.70729)
Supplement: Supplementary file 2 [file BJH-209-1907-s008.docx]

**Supplementary Figures**

**Supplemental Figure 1**. **(A)** Monotherapy dose-response curves for all four AML cell lines following treatment with increasing concentrations of Venetoclax (VEN) for 48 hours or 5-azacytidine (AZA) and Decitabine (DAC) for seven days. Cell viability was assessed by Alamar Blue assay. Mean and standard error of the mean of triplicates for each cell line are shown. **(B)** Overview of statistical difference between cell lines for the different treatment. P-values were computed using Wilcoxon Rank Sum Tests and adjusted using Benjamini-Hochberg. Significantly adjusted p-values are highlighted in red. **(C)** Monotherapy dose-response curves for treatment with increasing concentrations of VEN for 24 hours, AZA, DAC, cytarabine (ARA-C), and GSK-3685032 (GSK5032) for three days (and seven days for GSK5032) in all four AML cell lines. Cell viability was determined using Alamar Blue assay. Mean and standard error of the mean from triplicate measurements are shown for each treatment and concentration.

**Supplemental Figure 2.** (A-E) Dose response matrixes for VEN-AZA, VEN-DAC, VEN-ARA-C and VEN-GSK5032 in Kasumi-1 cells. AZA, 5-azacytidine; ARA-C, Cytarabine; DAC, Decitabine; GSK5032, GSK-3685032; VEN, Venetoclax.

**Supplemental Figure 2.** **(A-E)** Dose response matrices for VEN-AZA, VEN-DAC, VEN-ARA-C and VEN-GSK5032 in Kasumi-1 cells. AZA, 5-azacytidine; ARA-C, Cytarabine; DAC, Decitabine; GSK5032, GSK-3685032; VEN, Venetoclax.

**Supplemental Figure 3.** **(A-E)** Dose response matrices for VEN-AZA, VEN-DAC, VEN-ARA-C and VEN-GSK5032 in KG-1a cells. AZA, 5-azacytidine; ARA-C, Cytarabine; DAC, Decitabine; GSK5032, GSK-3685032; VEN, Venetoclax.

**Supplemental Figure 4.** **(A-E)** Dose response matrices for VEN-AZA, VEN-DAC, VEN-ARA-C and VEN-GSK5032 in MOLM-13 cells. AZA, 5-azacytidine; ARA-C, Cytarabine; DAC, Decitabine; GSK5032, GSK-3685032; VEN, Venetoclax.

**Supplemental Figure 5.** **(A-E)** Dose response matrices for VEN-AZA, VEN-DAC, VEN-ARA-C and VEN-GSK5032 in THP-1 cells. AZA, 5-azacytidine; ARA-C, Cytarabine; DAC, Decitabine; GSK5032, GSK-3685032; VEN, Venetoclax.

**Supplemental Figure 6.** **(A-D)** 3D surface plots depicting synergy scores for the combination effects of VEN-AZA, VEN-DAC, VEN-ARA-C, and VEN-GSK5032 across all four AML cell lines. Synergy scores were calculated using the Bliss Independence model. Mean Bliss score and the corresponding p-values are shown above each plot. AZA, 5-azacytidine; ARA-C, Cytarabine; DAC, Decitabine; GSK5032, GSK-3685032; VEN, Venetoclax.

**Supplemental Figure 7. (A)** Comparison of observed combination effects to the expected effects calculated using the ZIP synergy model. Lines connect the expected and observed values for each dose combination; red indicates potential synergy and blue potential antagonism. Statistical differences between the expected and observed effects were assessed using the Wilcoxon Rank Sum Test. **(B)** ZIP synergy scores for VEN combinations with AZA, DAC, ARA-C, or GSK5032, show as heatmaps (three replicates per condition). **(C)** Mean ZIP synergy score and significance for each VEN combination (AZA, DAC, ARA-C, GSK5032) divided per cell line. * = p < 0.05, ** = p < 0.01, *** = p < 0.001, and **** = p < 0.0001. AZA, 5-azacytidine; ARA-C, Cytarabine; DAC, Decitabine; GSK5032, GSK-3685032; VEN, Venetoclax.

**Supplemental Figure 8.** **(A-D)** 3D surface plots depicting synergy scores for the combination effects of VEN-AZA, VEN-DAC, VEN-ARA-C, and VEN-GSK5032 across all four AML cell lines. Synergy scores were calculated using the ZIP synergy model. Mean ZIP score and the corresponding p-values are shown above each plot. AZA, 5-azacytidine; ARA-C, Cytarabine; DAC, Decitabine; GSK5032, GSK-3685032; VEN, Venetoclax.

**Supplemental Figure 9.** Expression of the methylation-sensitive genes *DAZL* and *GAGE12* was measured by qPCR in KG-1a and MOLM-13 cells after treatment with increasing concentrations of 5-azacytidine (AZA), decitabine (DAC), and GSK-3685032 (GSK5032) for three days. Relative differences in expression compared to the housekeeping gene *GAPDH*.

**Supplemental Figure 10.** Viability of THP-1 cells following GSK-3685032 (GSK5032) pre-treatment and venetoclax (VEN) exposure. THP-1 cells were pretreated with 100 nM GSK5032 or DMSO for 72 hrs, then seeded at 10, 100, or 1000 cells per well and cultured for 14 days. Cells were subsequently treated with 10 nM venetoclax (VEN) or DMSO for 24 hrs. Viability was assessed using the Alamar Blue assay. The bars represent mean of six replicate wells per condition.
